# A comparative 48 month randomized trial of clinical performance and wear of BISGMA based and BISGMA free nanoceramic resin composites

**DOI:** 10.1038/s41598-025-16865-x

**Published:** 2025-08-25

**Authors:** Samah Mohamed Bahig, Heba Helal El Sherbiney, Mohamed Moustafa Zayed, Shereen Hafez Ibrahim

**Affiliations:** 1 Faculty of Dentistry, SinaiUniversity Kantra branch, Sinai, Egypt; 2 Conservative Dentistry Department, Faculty of Dentistry, Cairo University, Giza, Egypt

**Keywords:** Modified USPHS, Nanoceramic, Neo spectra, Occlusal wear, Resin composite restoration, Wear resistance, Zenit, Composite resin, Bonded restorations

## Abstract

This study aimed to compare the 48-month clinical performance and wear of Bis-GMA-based and Bis-GMA-free nanoceramic resin composites in Class I posterior restorations. In a randomized clinical trial, 64 patients received occlusal restorations with either Zenit (Bis-GMA-based) or Neo Spectra ST (Bis-GMA-free) nanoceramic composites (*n* = 32). Clinical performance was evaluated using modified USPHS criteria at four timepoints (baseline, 12, 24, 48 months). Intraoral scans were analyzed using 3D digital superimposition techniques to assess linear and volumetric quantification of wear across follow-up periods. The results revealed that marginal discoloration was slightly more frequent in the Zenit group at 48 months, though not statistically significant. Clinical outcomes were comparable between groups. The amount of linear deviation measured in Zenit samples was higher than in Neo Spectra, whereas the volumetric deviation was greater in Neo Spectra. However, neither difference was statistically significant. Both composites demonstrated clinically acceptable performance over a 48-month period in Class I posterior restorations. Some marginal discoloration was observed with both materials. The differing matrix-to-filler ratios of the two nanoceramic resin composites may have contributed to compensating for volumetric wear. Intraoral scanning and digital analysis enable accurate, non-invasive wear monitoring. Neo Spectra ST offers superior esthetic stability and clinical handling. Neo Spectra™ ST may offer a clinically advantageous option for posterior restorations requiring esthetic durability and operator-friendly handling. Additionally, digital intraoral scanning combined with registration software provides a promising, non-invasive approach for monitoring restorative wear in clinical practice.

**Clinical trial registration**: This study was registered on clinical trial (http://www.ClinicalTrials.gov) at February 4, 2021 with ID: NCT04738604.

## Introduction

Wear resistance is one of the most significant mechanical properties of resin composites and plays a major role in the clinical durability of dental restorations. Ideally, the wear characteristics of restorative materials should approximate those of natural teeth^1^. Despite continuous improvements in composite resin formulations, wear remains a primary cause of restoration failure, with estimates suggesting that approximately 77 million posterior resin composite restorations will be affected by noticeable wear, and over 30 million placed in 2015 may require repair or replacement by 2025 due to wear-related issues^2,3^.

The development of alternative monomer systems to Bis-GMA has become essential to address its limitations related to durability and potential cytotoxicity. One such alternative is the incorporation of hybrid organoceramic components into methacrylate-based resin matrix composites, known as organically modified ceramic (ORMOCER^®^, VOCO GmbH, Cuxhaven, Germany), a subclass of organically modified silicates (ORMOSILs). A key advantage of ORMOCER^®^ materials lies in their unique hybrid network, which combines polysiloxane backbones with photopolymerizable methacrylate groups that are covalently bonded to silica-based fillers. This molecular architecture replaces traditional oxygen linkages with organic functional groups, resulting in a densely cross-linked, three-dimensional polymer network. Compared to conventional resin composites, ORMOCER^®^-based materials contain a lower proportion of organic matrix, contributing to improved biocompatibility through reduced residual monomers, minimized polymerization shrinkage, enhanced wear resistance, increased opacity, and superior handling characteristics^4^.

The wear behavior of resin composites is influenced by several factors, including resin matrix composition, filler size and content, and the strength of the filler–matrix bond. Smaller filler particles, higher filler loading, and improved polymerization are associated with enhanced wear resistance, reduced surface roughness, and extended clinical longevity^5–7^.

Clinical wear of restorative materials has traditionally been assessed through direct visual examination or indirect measurement from dental casts, but both methods have limitations in detecting small-scale wear^8^. Intraoral scanning (IOS) has recently emerged as a valuable tool that provides non-invasive, high-resolution digital data, enabling accurate and quantitative wear analysis through 3D superimposition software^5^.

The use of intraoral scanners (IOS) with best-fit superimposition techniques has increasingly been verified for tracking occlusal wear and development in clinical trials. Digital methods offer non-invasive, high-resolution monitoring of surface changes at greater accuracy and replicability than traditional impressions. Studies have found that 3D superimposition of intraoral scans is able to quantify occlusal wear accurately even when considerable morphological change occurs on the other tooth surfaces, which proves best-fit stability under challenging clinical conditions. Additionally, clinical trials comparing antagonist enamel wear with restorative materials have once more highlighted the need for precise, standardized assessment methods in long-term wear studies. These results lend support to the method used in this study, validating the clinical utility of IOS-based linear and volumetric wear analysis as measures to assess restorative materials longitudinally^9,10^.

Although wear resistance is essential for the long-term success of dental restorations, resin composite materials continue to exhibit lower wear resistance compared to natural enamel^8^. Furthermore, limited long-term clinical data exist on the occlusal wear of nanoceramic composites assessed using IOS^7^. Although composites with nano-sized ceramic fillers are referred to as “nanoceramic,” the term is not standardized and does not ensure consistency in composition or clinical behavior^3^. Although both Zenit (Bis-GMA-based) and Neo SpectraTM ST (UDMA-based, Bis-GMA-free) are categorized as nanoceramic composites, their filler technology and resin chemistry differ significantly. Few long-term comparative studies have assessed their mechanical performance, handling, and aesthetic results under standardized conditions, despite their extensive clinical use^3,11^.

Although most available clinical studies of nanoceramic materials are related to indirect restorations, their favorable clinical behavior has been disclosed by recent systematic reviews. Sharing resin matrix technologies and nanofiller structures with direct nanoceramic composites, these materials have shown high survival rates, acceptable marginal adaptation, and acceptable wear resistance. As an example, survival rates above 90% after 3 years and 84% after 5 years have been reported for resin nanoceramic and resin-matrix ceramic restorations in posterior teeth. Such findings, while based on CAD–CAM or partial coverage indications, provide valuable context and support the overall stability and long-term performance of nanoceramic materials for restorative dentistry^12,13^.

The aim of this randomized clinical trial was to compare the clinical performance and occlusal wear of these two nanoceramic resin composites in Class I posterior restorations using intraoral digital scanning. The goal was to provide evidence-based guidance to assist clinicians in material selection. We hypothesized that there would be no significant differences in clinical outcomes or wear between the two materials over the 48-month period.

## Materials and methods

The materials used in this randomized clinical trial were Zenit nano - Ceramic Composite (President Dental; Munich, Germany) and Neo Spectra ST Universal Composite (Dentsply DE Trey, Konstanz, Germany). Zenit, although marketed as “nanoceramic” due to the inclusion of fine radiopaque porcelain fillers, Zenit lacks documented evidence of advanced filler engineering and instead appears to rely on a more traditional nano-hybrid configuration with randomly distributed or bimodal nanofillers, whereas SphereTEC was an accurate and developed granulated filler technology composed of pre-polymerized spherical clusters with nano-sized filler particles, is used by Neo Spectra™ ST who published studies and precise microstructure manufacturer data.

Zenit is a conventional resin matrix (Bis-GMA or Bis-EMA based), whereas Neo Spectra™ ST has a Bis-GMA-free matrix based on UDMA. All materials used as well as their descriptions, principal components and manufacturers are listed in Table 1.

**Table 1:** Material name, lot number, specification, manufacturer and composition.

Material name	Specification	Composition	Manufacturer	Lot number
President dental etching gel	Etchant	37% phosphoric acid solution	President Dental; Munich, Germany	#PD9D0237
Prime and bond universal	Light cured universal adhesive	PENTA, 10MDP, Active GuardTM Technology crosslinker, isopropanol, water, initiator, stabilizer	Dentsply De Trey GmbH, Konstanz, Germany	#2,101,001,077
Zenit	Light cured, universal nanoceramic composite	UDMA, Butanediol MA, Bis GMA. Pyrogenic silica 12 nm Glass filler. 7 μm Agglomerated nanoparticles 0.6 μm. The total filling weight is 83% by (70% in volume)	President Dental; Munich, Germany	#2,019,009,766
Neo Spectra™ ST	Light cured, universal nanoceramic composite	Methacrylate modified polysiloxane (organically modified ceramic) Di methacrylate resins, ethyl-4 (dimethylamine) benzoate, and bis(4-methyl-phenyl) iodonium hexafluorophosphate. Filler load: 78–80% by weight (57% by volume): Spherical, pre-polymerized Sphere TEC fillers (d3,50 ≈ 15 μm), non-agglomerated barium glass and ytterbium fluoride	Dentsply, Konstanz, Germany	#2,102,001,020
Dynamic flow	A low viscosity, visible light curing flowable resin composite	BIS-GMA, UDMA.Bis-Ema TMPTMA Barium aliminium boro silicate	President Dental; Munich, Germany	#2,019,009,766

### Trial registration and ethical approval

The protocol of this study was registered on clinical trial (http://www.ClinicalTrials.gov) on 4/2/2021 with ID: NCT04738604. All participants were given an informed consent form approved by the faculty’s ethics committee (approval number N91120). with Sample size approval from Medical Biostatistics Unit (MBU).

### Sample size calculation

We conducted a power analysis to ensure sufficient statistical power to test the null hypothesis that no significant difference would be found between the tested groups in terms of clinical success rate. An alpha (α) level of 0.05, a beta (β) level of 0.2 (i.e., power = 80%), and an effect size (Ꞷ) of 0.386—calculated based on the results of a previous study^11^. Based on these parameters, the total required sample size was calculated to be 54 cases. To account for potential dropouts during the follow-up period, the sample size was increased by 20%, resulting in a final sample of 64 cases (32 cases per group). We performed the sample size calculation using R statistical analysis software, version 4.4.2 for Windows^14^.

### Eligibility criteria

Patients with vital posterior first or second molars presenting with Class I carious lesions (ICDAS II scores 3 or 4) were eligible for inclusion in the trial if they met all of the following criteria: age between 20 and 54 years, cooperative behavior, and willingness to participate in the study. Tooth-specific inclusion criteria included cavity preparations with no undermined enamel walls and a minimum remaining wall thickness of 2 mm.

All lesions were initially classified as ICDAS II scores 3 or 4. Cavity preparation was performed using a conservative caries removal technique, whereby demineralized tissue was selectively removed until firm dentin was reached, based on tactile and visual assessment. This approach occasionally resulted in localized dentin exposure; however, no cases exhibited progression to deep cavitations consistent with ICDAS score 5.

The included tooth had to be functional and have an intact opposing tooth. Exclusion criteria included molars with pulpal and/or periodontal pathology, patients with medical complications, teeth that were mobile or loose, teeth with severe wear or significant erosion, non-functional teeth lacking opposing occlusion, and patients with bruxism habits.

### Study design and grouping

This trial was conducted at the outpatient clinic of the Faculty of Dentistry, Cairo University, between February 2021 and February 2025. It was a 48-month, double-blinded, randomized clinical trial with two parallel arms and a 1:1 allocation ratio. A total of 64 patients (35 males and 29 females) with occlusal carious lesions were included and randomly assigned to two groups, with 32 teeth in each group. The intervention group (A1) received Zenit nanoceramic composite restorations, while the control group (A2) received Neo Spectra ST Universal composite restorations. All participants were informed about the study objectives and potential risks and provided written informed consent prior to inclusion.

### Randomization, allocation concealment, and blinding

**Fig. 1:**
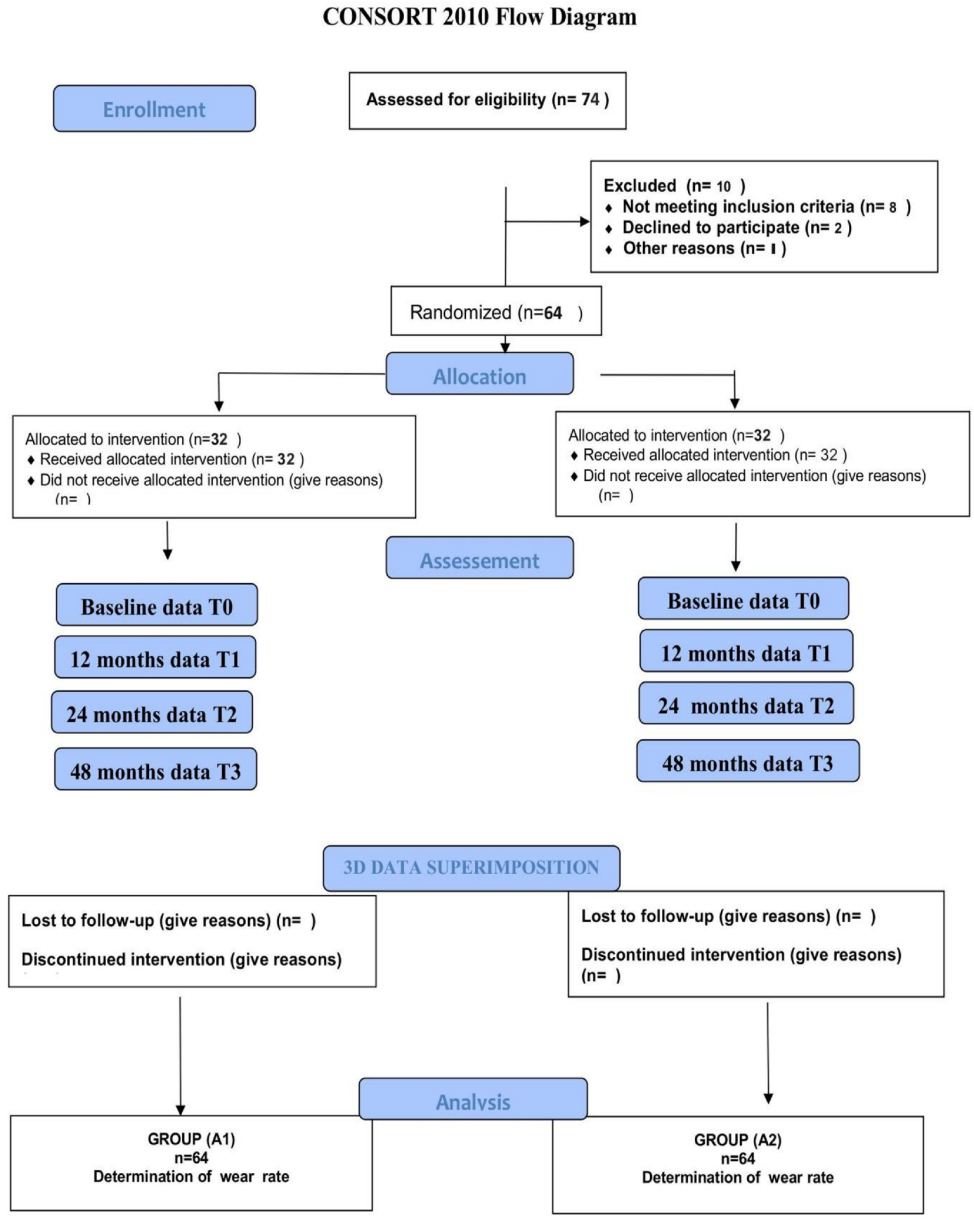
Flowchart of the study.

Sixty-four participants were randomly allocated into two groups using online randomization software (https://www.randomizer.org/). Random numbers from 1 to 32 represented the intervention group, while numbers from 33 to 64 represented the comparator group. Both participants and clinical evaluators were blinded to group allocation to minimize assessment bias. Randomization codes were generated and placed in sealed, opaque, sequentially numbered envelopes, which were opened only at the time of restoration placement. This ensured that the operator was informed of the assigned material immediately before the procedure, allowing adherence to the manufacturer’s protocol. During all follow-up visits, two independent, calibrated evaluators, blinded to group assignment, performed outcome assessments. A summary of the trial process is presented in the CONSORT 2010 flow diagram (Fig. 1).

### Field preparation and restorative procedures

The operator obtained a periapical radiograph for every tooth using an intraoral periapical sensor (EzSensor HD, VATECH, Gyeonggi-do, Korea) prior to enrollment. These radiographs were used to evaluate pulpal and periapical status, confirm the absence of pathology, and ensure eligibility for inclusion. As only occlusal Class I lesions were included, caries diagnosis was based on clinical examination using ICDAS II criteria, and interproximal radiographs were not routinely indicated.

Each tooth was isolated with a rubber dam. A standardized Class I cavity was prepared, limited to a maximum of one-third of the intercuspal distance, using Bur (No. 2455, Jota AG, Switzerland) in a high-speed handpiece (Pana-Air, NSK, China) with copious water irrigation. The operator removed carious tissue until firm dentin was reached using a sterile, low-speed round carbide bur(MANI, INC, Japan) attached to a contra-angle low-speed handpiece (WE-56 T, W&H, Austria) under irrigation, and supplemented by a sharp excavator (Zeffiro, Lascod Excavator Double-Ended, Italy). Cavity depth was measured using a periodontal probe (Nordent Manufacturing Inc., USA) and found to be 4 ± 0.3 mm, measured from the occlusal cavosurface margin to the gingival floor.

The operator carried out the restorations according to the manufacturers’ instructions. The operator applied 37% phosphoric acid gel (President Dental; Munich, Germany) to the enamel margins for 15 s using a selective etching technique. The operator then rinsed the cavity with an air-water spray for 30 s and gently dried it to avoid desiccating the dentin.

The etched enamel and dentin surfaces were treated with a universal adhesive, Prime&Bond Universal (Dentsply DeTrey, Konstanz, Germany). The operator applied the adhesive in two separate coats using a microbrush (Ivoclar Vivadent), with each coat vigorously rubbed for 20 s. A gentle air stream was then applied for 10 s to evaporate the solvent. The adhesive was subsequently light-cured for 20 s using an LED curing unit with a light intensity of up to 1600 mW/cm² (Radii Plus, SDI Limited, Bayswater, Victoria, Australia). The curing unit’s light intensity was monitored and verified after every 10 uses using the built-in radiometer of the Radii Plus device.

The intervention group (A1) included a total of 32 teeth. A flowable composite layer approximately 0.5 mm thick (Dynamic Flow, President Dental; Munich, Germany) was applied to the cavity floor and light-cured for 20 s. The remaining cavity was restored using ZENIT nano-ceramic composite (President Dental; Munich, Germany) with an oblique incremental technique, placing increments no thicker than 2 mm. Each increment was light-cured from the occlusal surface for 20 s.

In the comparator group (A2), a total of 32 teeth were restored. A 0.5 mm layer of flowable composite (Dynamic Flow, President Dental; Munich, Germany) was applied to the cavity floor and light-cured for 20 s. Neo Spectra™ ST (Dentsply DeTrey GmbH, Konstanz, Germany) was then placed using an incremental technique, with each layer not exceeding 2 mm in thickness. Then light-cured each increment for 20 s, following the manufacturer’s instructions. The use of a flowable composite liner in both groups aimed to enhance adaptation, minimize void formation, and reduce polymerization stress, particularly in the high C-factor environment of Class I cavities. Occlusal prematurities in both centric and eccentric positions were checked using 40 μm carbon articulating paper (AccuFilm^®^ II, Parkell^®^, Edgewood, NY, USA). The operator performed finishing and polishing of all restorations under water cooling using superfine yellow tapered and football-shaped diamond stones (Coltene Whaledent, Switzerland). Final polishing was completed using a composite polishing kit (One Gloss PS, SHOFU INC, Japan) and a felt wheel with aluminum oxide polishing paste.

### Clinical evaluation of the restoration (1ry outcome assessment)

Two blinded investigators assessed the clinical performance of the restorations using the modified US Public Health Service (USPHS) criteria^15^ (Tables 2 and 3). To assess intra-rater reliability, every examiner revisited 20% of the cases after a two week period. Cohen’s kappa values were recorded as 0.89 and 0.91, meaning there was nearly perfect consistency. Between the two examiners, inter-rater reliability produced a kappa value of 0.87, further supporting a strong level of agreement^16^.

**Table 2:** Modified USPHS criteria.

Criteria/score	Alpha (A)	Bravo (B)	Charlie (C)	Delta (D)
Anatomic contour (integrity filling) (visual inspection and explorer)	The restoration is a continuation of existing anatomic form or is slightly flattened. It may be over-contoured When the side of the explorer is placed tangentially across the restoration, it does not touch two opposing cavo-surface line angles at the same time	A surface concavity is evident. When the side of the explorer is placed tangentially across the restoration, it does not touch two opposing cavosurface line angles at the same time, but the dentin or base is not exposed	There is a loss of restorative substance such that a surface concavity is evident, and the base and/or dentin is exposed	Total loss of the restoration
Marginal adaptation (visual inspection and explorer)	The explorer does not catch when drawn across the surface of the restoration toward the tooth, or, if the explorer does not catch, there is no visible crevice along the periphery of the restoration	The explorer catches and there is visible evidence of a crevice, which the explorer penetrates, indicating that the edge of the restoration does not adapt closely to the tooth structure. The dentin and/or the base is not exposed, and the restoration is not mobile	The explorer penetrates crevice defect extended to the dentino- enamel junction. (localized)	Strong negative step in major parts of the margin not removable
Surface texture (explorer)	Surface texture similar to polished enamel as determined by means of a sharp explorer	Surface texture gritty or similar to a surface subject to a white stone or similar to a composite containing supra-micron sized particle	Surface pitting is sufficiently coarse to inhibit the continuous movement of an explorer across the surface	
Gross fracture	Restoration is intact and fully retained	Restoration is partially retained with some portion of the restoration still intact	Restoration is completely missing	
Cavo-surface marginal discoloration (visual inspection)	There is no visual evidence of marginal discoloration different from the color of the restorative material and from the color of the adjacent tooth structure	There is visual evidence of marginal discoloration at the junction of the tooth structure and the restoration, but the discoloration has not penetrated along the restoration in a pulpal direction	There is evidence of visual marginal discoloration at the junction of the tooth structure and the restoration that has penetrated along the restoration in a pulpal direction	Strong discoloration in major parts Of the margins, not removable
Color match (visual inspection)	The restoration matches the shade and translucency of the adjacent tooth	There is a mismatch in the shade and translucency, but it is within the normal range of tooth shade	The mismatch beyond the normal range of the tooth shade and translucency	
Secondary caries (visual inspection)	The restoration is a continuation of existing anatomic form adjacent to the restoration	There is visual evidence of dark keep discoloration adjacent to the restoration (but not directly associated with cavo- surface margins)		
Postoperative hypersensitivity	No postoperative sensitivity, after the restorative procedure and during the study		Sensitivity at any stage of the study	

**Table 3:** Prioritization of outcomes.

Prioritization of outcome	Outcome	Method of measure	Unit of measurement
Primary outcome: (Modified USPHS)	Anatomic form	Clinical evaluation	Alfa: Anatomic form ideal
			Bravo: Restoration is under-contoured, without dentin or base exposure.
			Charlie: Restoration is under-contoured, without dentin or base exposure.restoration need replacement
Secondary outcomes	Color match	Clinical evaluation	Alfa: Matches tooth
			Bravo: Acceptable mismatch
			Charlie: Unacceptable mismatch
	Marginal discoloration	Clinical evaluation	Alfa: No discoloration
			Bravo: minor marginal discoloration without staining toward pulp
			Charlie: Deep discoloration with staining toward pulp
	Marginal adaptation	Clinical evaluation	Alfa: Closely adapted, no visible crevice
			Bravo: Visible crevice, explorer will penetrate
			Charlie: Crevice in which dentin is exposed
	Secondary Caries	Clinical evaluation	Alfa: No active caries present
			Bravo: Non-cavitated active caries is present in contact with the restoration
			Charlie: Cavitated active caries is present in contact with the restoration
	Surface Texture	Clinical evaluation	Alfa: As smooth as the surrounding enamel
			Bravo: Surface rougher than enamel, clinically acceptable
			Charlie: Surface unacceptably rough
	Marginal Integrity	Clinical evaluation	Alfa: Restoration adapts closely to the tooth structure
			Bravo: A visible crevice
			Charlie: The explorer penetrates into the crevice
	Postoperative sensitivity	Clinical evaluation	Alfa: No post-operative sensitivity
			Bravo: Short term and tolerable post-operative sensitivity
			Charlie: Intolerable post-operative sensitivity
Amount of restoration wear (2ry outcome)		A-Digital scanner (Geometrical Subtraction software) B-Impression replica technique (Nikolaos et al., in 2020)	Measuring unit: (Quantitative) Linear deviation (mm) Volumetric deviation (mm^3^)

### Wear quantification (2nd outcome assessment)

Patients were scanned at four different time points: immediately after restoration placement (T0), and at 12 months (T1), 24 months (T2), and 48 months (T3). To ensure that the initial scan accurately represented the final clinical form of the restoration, the T0 baseline scan was acquired after finishing, polishing, and any necessary occlusal adjustments.

Descriptive statistics for tooth wear were calculated from the scans of 64 patients, focusing on linear deviation (mm) and volumetric deviation (mm³) between the T0–T1, T0–T2, and T0–T3 intervals to evaluate the magnitude and clinical relevance of wear over time^7^.

### Steps of wear quantification

#### Data acquisition

After completing adhesive bonding and occlusal adjustments of the restorations, the operator isolated the dentition using lip retractors and cotton rolls, then air-dried the area to prevent fluid pooling on the occlusal surfaces. Only the restored tooth and the mesial and distal adjacent teeth were scanned at baseline and during recall visits, according to the manufacturer’s instructions, using an intraoral scanner (Helios 600, Eighteeth, Changzhou Sifary Medical Technology Co. Ltd., China).

A single examiner exclusively performed the scans, calibrating the scanner before each use in accordance with the manufacturer’s protocols and conducting internal calibration procedures to ensure consistency in scan acquisition and data processing. To maintain high data quality, only scan alignments with overlay errors below 15 μm (initial registration) and 30 μm (final alignment) were accepted for analysis.

The examiner cleaned and firmly secured the scanner tip to ensure optimal access and visibility and performed the scans with the patient seated in an upright position and the teeth positioned as close to the scanner tip as possible. Scanning began at the occlusal surface, followed by the palatal/lingual and finally the buccal surfaces of the upper and lower teeth, respectively^5^. To assess intra-operator reliability, the linear and volumetric wear measurements for the ten randomly selected scan datasets were computed using intraclass correlation coefficients (ICCs). With two weeks between sessions where the same examiner rescaned and reprocessed the datasets, results showed excellent repeatability (ICC = 0.93 for linear deviation and 0.91 for volumetric deviation). Due to the sole use of a single examiner for scanning and processing in this study, inter-operator reliability was not relevant^17^.

#### Processing of datasets

A single calibrated examiner imported the STL datasets generated from the baseline and follow-up scans into 3D metrology software (Geomagic Control X 2022) and conducted the analysis by isolating individual digital models of the restored first molars and saving them as separate datasets for restoration-specific evaluation.

Data points below the tooth equator were removed. A best-fit algorithm was then used to superimpose the follow-up data onto the baseline data, initially aligning the entire restoration surface of the reference and recall datasets. The average overlay error was calculated after visual confirmation of alignment quality. Only surfaces with deviations smaller than the calculated overlay error were included in the subsequent best-fit refinement. The calibrated examiner repeated this process iteratively until the overlay error reached a minimum and no longer decreased. The examiner included only datasets (COMP *n* = 39) with final overlay errors below 15 μm in the final analysis^4^.

#### Superimposition

The calibrated examiner superimposed the recall data onto the baseline data using an iterative best-fit method. The algorithm aligned the pre and post-wear datasets by establishing stable reference regions unaltered areas such as tooth surfaces—to minimize alignment errors. A visual assessment of the superimposition outcome was conducted to verify alignment accuracy and calculate the average overlay error. A refined best-fit alignment was then applied, excluding regions with the largest deviations^7^.

**Fig. 2:**
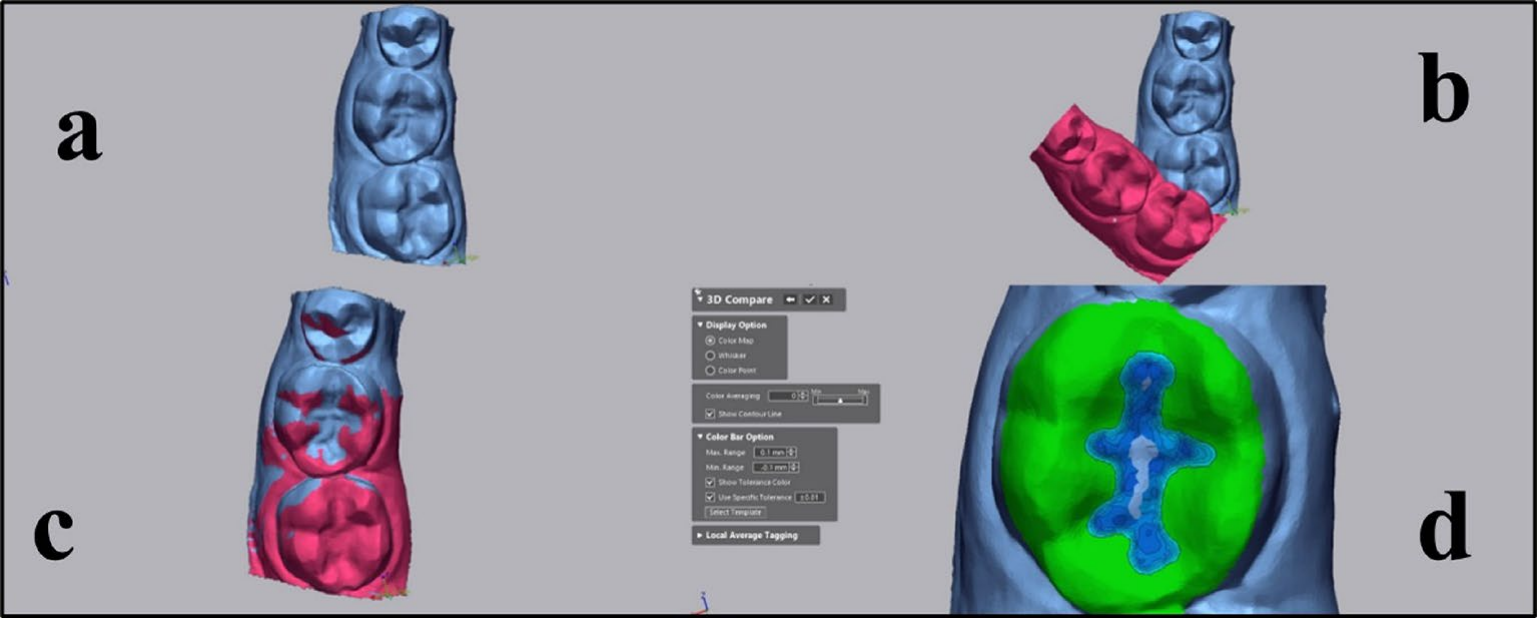
Teeth scanned using 3D metrology software (Geomagic Control X 2022) showing Visual representation of the steps for superimposition and quantitative wear measurement **(a)** Import of baseline scan. **(b)** Comparison of measured and reference data. **(c)** Automatic best-fit alignment minimizing surface deviation. **(d)** 3D color mapping and contour visualization to identify wear areas.

The alignment process was repeated iteratively until no further reduction in overlay error was observed. Only datasets with a final overlay error below 0.03 mm were included for analysis. Color-coded superimposed models were then used to visualize and localize wear on the restorations caused by opposing dentition. Only regions exhibiting detectable wear indicated by blue on the heatmap were considered for further analysis^5^ (Fig. 2).

### Measurement of linear deviation

Linear deviation measurement was used to quantify material loss or changes in wear depth between two 3D datasets. Using the 3D comparison tool, the occlusal surface was selected for analysis, and the maximum negative deviation value was extracted to represent the deepest point of wear.

### Measurement of volumetric deviation

**Fig. 3:**
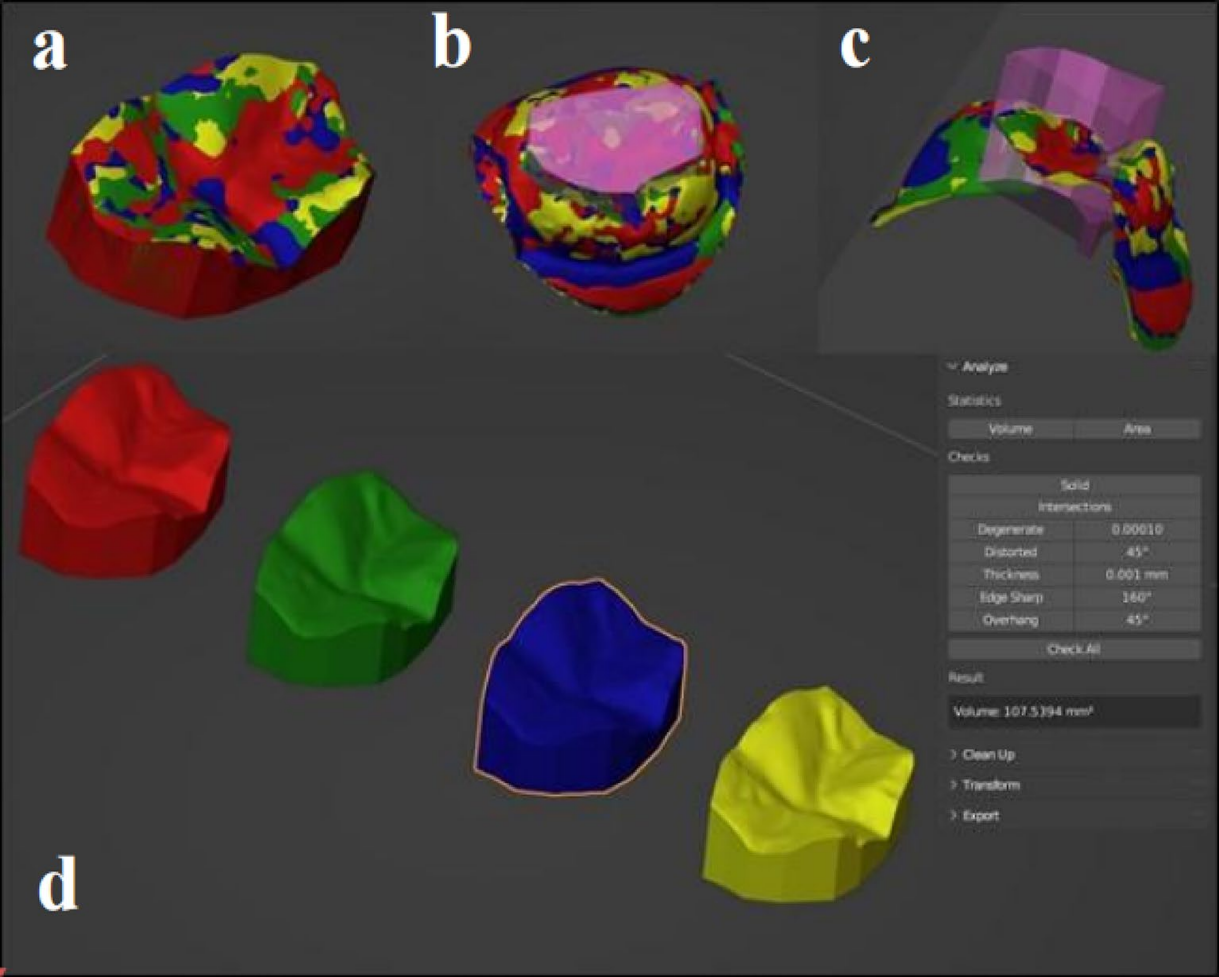
Teeth scanned using 3D metrology software (Geomagic Control X 2022) showing Occlusal surface extraction and wear analysis. **(a)** Color-coded 3D models of occlusal surfaces at different recall periods. **(b)** Isolation of the occlusal table using intersecting volume (pink prism). **(c)** Cross-sectional view showing surface changes and prism penetration depth. **(d)** Separated 3D volumes with corresponding measurements at each time point (T0, T1, T2, T3). Each color-coded (red, green, blue, and yellow) corresponding to T0, T1, T2 and T3 respectively for ease of differentiation.

The calibrated examiner used volumetric deviation measurement to quantify material loss or changes in total volume between the two 3D datasets, then exported the superimposed datasets from the metrology software while preserving their aligned coordinates and imported them into an open-source 3D modeling program (Blender v3.9 LTS). To quantify volumetric wear, the occlusal surface was digitally divided and its volume at each recall visit was quantified using 3D modeling software. Follow-up to baseline volume difference was measured in cubic millimeters (mm³) and recorded for analysis^7^ (Fig. 3).

### Statistical analysis

Categorical data (demographics) and ordinal data (clinical scores) were presented as frequencies and percentages. Numerical data (deviation measurements) were presented as mean, standard deviation (SD), median, and interquartile range (IQR). Normality was assessed using distribution plots and the Shapiro–Wilk test and indicated that the data were non-parametric. Categorical variables were analyzed using Fisher’s exact test. Ordinal and numerical variables were analyzed using the Mann–Whitney U test and Friedman’s test, followed by the Nemenyi post hoc test for intergroup and intragroup comparisons, respectively. P-values were adjusted for multiple comparisons using the False Discovery Rate (FDR) method. Cohen’s ω was reported as an effect size for the comparisons of categorical data, while Rank-biserial correlation (r_rb_) and Kendall’s W were used for the comparisons of independent and repeated nonparametric data, respectively. Confidence intervals for effect sizes were calculated using bootstrapping. A significance level of *p* < 0.05 was adopted for all statistical tests. Statistical analysis was performed using R software version 4.4.2 for Windows (R Core Team, 2024. R: A language and environment for statistical computing. R Foundation for Statistical Computing, Vienna, Austria. https://www.R-project.org/^14^.

## Results

**Fig. 4:**
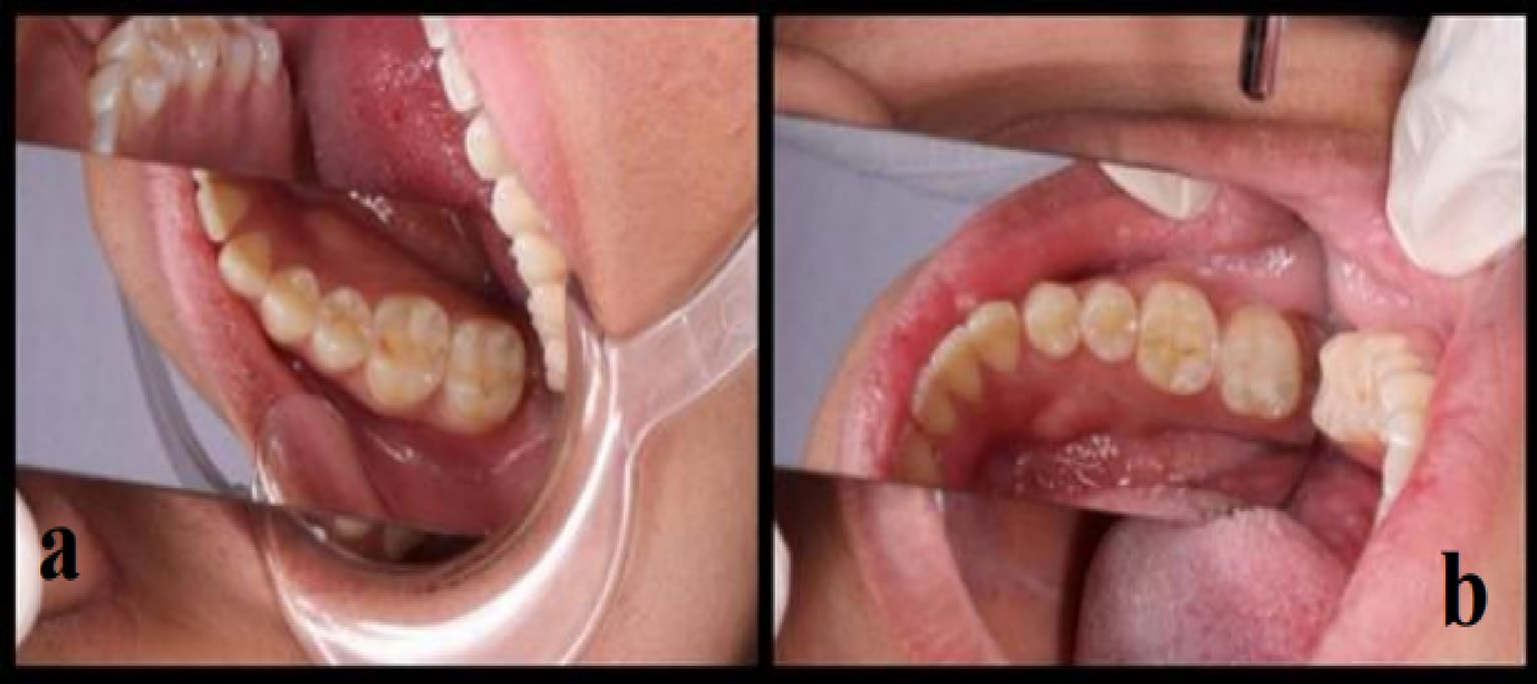
(**a**) Score B of marginal discoloration of Zenit in tooth #36 after a 48-month follow-up period, (**b**) Score B of marginal discoloration of Neo spectra in tooth #46 after a 48-month follow-up period.

**Fig. 5:**
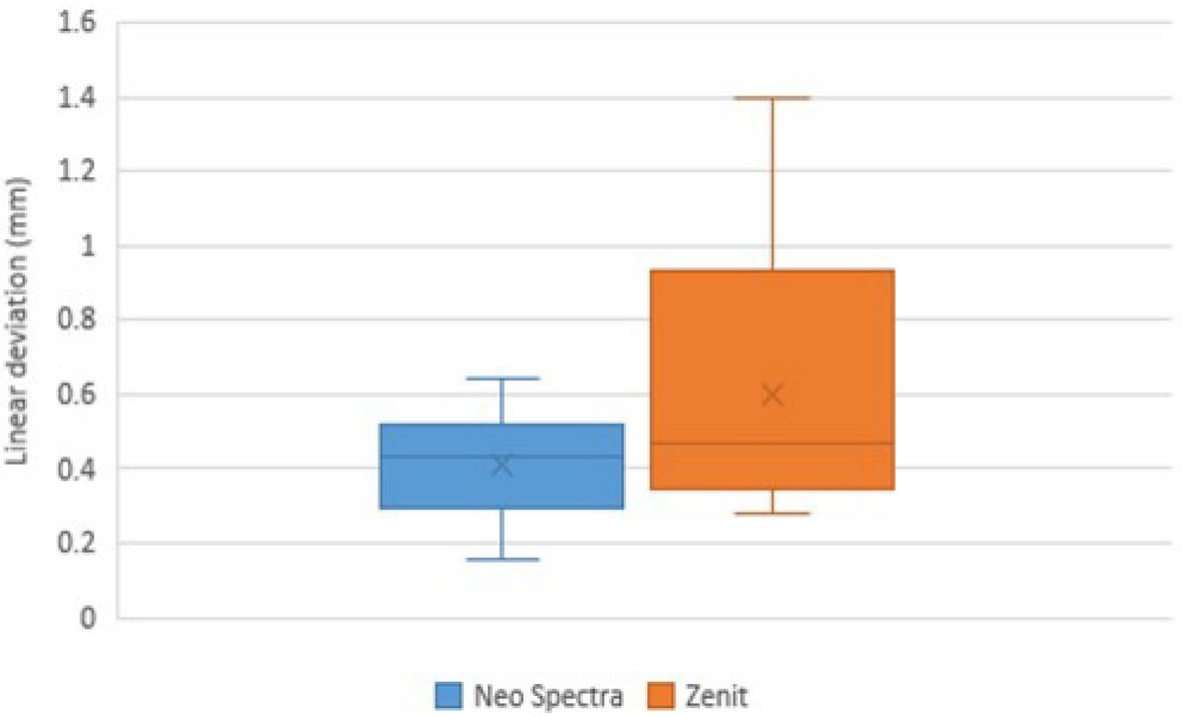
Box plot for linear deviation values.

**Fig. 6:**
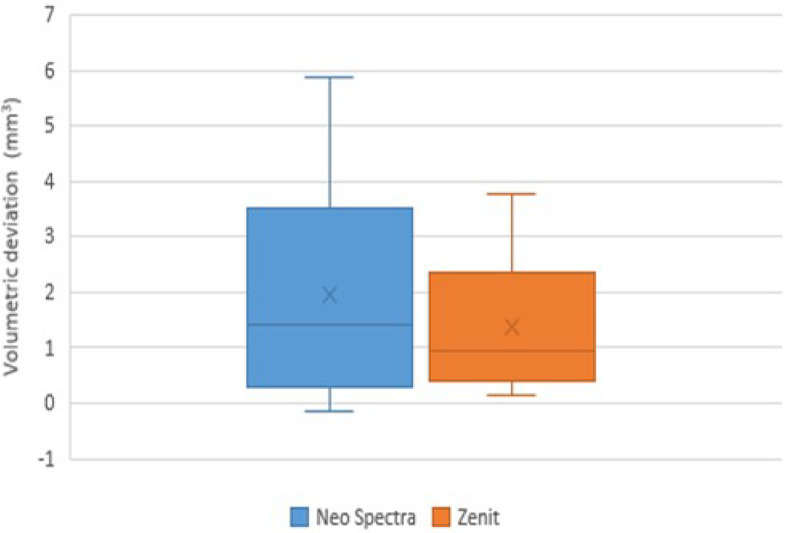
Box plot for volumetric deviation values.

The study included 64 cases, which were randomly and equally allocated into two groups. All 64 participants completed the study. Table 4 presents various demographic parameters, with no statistically significant differences observed between the groups, and all effect sizes were small. The distribution of clinical scores is shown in Tables 5 and 6. For marginal discoloration at the final interval (T3), two cases in the Neo Spectra group and four in the Zenit group had a (B) score with no statistically significant difference (*p* = 0.402) and a small effect size (r_rb_ = −0.06, 95% CI [−0.33, 0.22]) (Fig. 4). For all other parameters and intervals, all cases in both groups had an (A) score. Within the Zenit group, the percentage of cases with a (B) score for marginal discoloration at T3 was significantly higher than at other intervals (*p* = 0.014), with a small effect size (W = 0.12, 95% CI [0.03, 0.25]). However, the Neo Spectra group’s difference was not statistically significant (*p* = 0.112), and the effect size was similarly small (W = 0.06, 95% CI [−0.01, 0.16]). Table 7; Figs. 5 and 6 present the results of deviation measurements. They show that the amount of linear deviation measured in Zenit samples was higher than in Neo Spectra, whereas the volumetric deviation was greater in Neo Spectra. However, neither difference was statistically significant, and both were associated with small effect sizes. Although no significant differences were found in linear or volumetric deviation, effect size estimates offered additional perspective. In some cases, the confidence intervals were broad, which is an expected consequence of measurement variability rather than a limitation in study design, as the sample size was determined through a priori power analysis. There was no dropout rate because all 64 participants finished the 48-month follow-up. No intention-to-treat analysis was needed due to zero dropout.

**Table 4:** Demographic data.

Parameter		n (%)		Test statistic	p-value	Cohen’s ω (95% CI)
		Neo spectra	Zenit			
Sex	Male	21 (66%)	14 (44%)	3.09	0.134	0.22 (0.00 to 0.47)
	Female	11 (34%)	18 (56%)			
Treated arch	Lower	27 (84%)	30 (94%)	1.44	0.423	0.15 (0.00 to 0.40)
	Upper	5 (16%)	2 (6%)			
Treated side	Right	12 (38%)	16 (50%)	1.02	0.440	0.13 (0.00 to 0.37)
	Left	20 (62%)	16 (50%)			
Treated tooth	First molar	18 (56%)	16 (50%)	0.25	0.780	0.06 (0.00 to 0.30)
	Second molar	14 (44%)	16 (50%)			

*CI* Confidence interval.

**Table 5:** Clinical scores (A).

Time	Score	n (%)							
		Anatomic contour		Color match		Gross fracture		Marginal adaptation	
		Neo spectra	Zenit	Neo spectra	Zenit	Neo spectra	Zenit	Neo spectra	Zenit
T0	A	32 (100%)	32 (100%)	32 (100%)	32 (100%)	32 (100%)	32 (100%)	32 (100%)	32 (100%)
	B	0 (0%)	0 (0%)	0 (0%)	0 (0%)	0 (0%)	0 (0%)	0 (0%)	0 (0%)
	C	0 (0%)	0 (0%)	0 (0%)	0 (0%)	0 (0%)	0 (0%)	0 (0%)	0 (0%)
	D	0 (0%)	0 (0%)	0 (0%)	0 (0%)	0 (0%)	0 (0%)	0 (0%)	0 (0%)
T1	A	32 (100%)	32 (100%)	32 (100%)	32 (100%)	32 (100%)	32 (100%)	32 (100%)	32 (100%)
	B	0 (0%)	0 (0%)	0 (0%)	0 (0%)	0 (0%)	0 (0%)	0 (0%)	0 (0%)
	C	0 (0%)	0 (0%)	0 (0%)	0 (0%)	0 (0%)	0 (0%)	0 (0%)	0 (0%)
	D	0 (0%)	0 (0%)	0 (0%)	0 (0%)	0 (0%)	0 (0%)	0 (0%)	0 (0%)
T2	A	32 (100%)	32 (100%)	32 (100%)	32 (100%)	32 (100%)	32 (100%)	32 (100%)	32 (100%)
	B	0 (0%)	0 (0%)	0 (0%)	0 (0%)	0 (0%)	0 (0%)	0 (0%)	0 (0%)
	C	0 (0%)	0 (0%)	0 (0%)	0 (0%)	0 (0%)	0 (0%)	0 (0%)	0 (0%)
	D	0 (0%)	0 (0%)	0 (0%)	0 (0%)	0 (0%)	0 (0%)	0 (0%)	0 (0%)
T3	A	32 (100%)	32 (100%)	32 (100%)	32 (100%)	32 (100%)	32 (100%)	32 (100%)	32 (100%)
	B	0 (0%)	0 (0%)	0 (0%)	0 (0%)	0 (0%)	0 (0%)	0 (0%)	0 (0%)
	C	0 (0%)	0 (0%)	0 (0%)	0 (0%)	0 (0%)	0 (0%)	0 (0%)	0 (0%)
	D	0 (0%)	0 (0%)	0 (0%)	0 (0%)	0 (0%)	0 (0%)	0 (0%)	0 (0%)

No p-values were added since all are NA to keep the table simpler.

**Table 6:** Clinical scores (B).

Time point	Parameter	Score A (*n*, %)	Score B (*n*, %)	Score C (*n*, %)	Score D (*n*, %)
T0	Marginal discoloration (neo spectra)	32 (100%)	0 (0%)	0 (0%)	0 (0%)
	Marginal discoloration (Zenit)	32 (100%)	0 (0%)	0 (0%)	0 (0%)
	Postoperative sensitivity (both groups)	32 (100%)	0 (0%)	0 (0%)	0 (0%)
	Secondary caries (both groups)	32 (100%)	0 (0%)	0 (0%)	0 (0%)
	Surface texture (both groups)	32 (100%)	0 (0%)	0 (0%)	0 (0%)
T1	Same as T0 (no changes)				
T2	Same as T0 (no changes)				
T3	Marginal discoloration (neo spectra)	30 (94%)	2 (6%)	0 (0%)	0 (0%)
	Marginal discoloration (Zenit)	28 (88%)	4 (12%)	0 (0%)	0 (0%)
	Postoperative sensitivity (both groups)	32 (100%)	0 (0%)	0 (0%)	0 (0%)
	Secondary caries (both groups)	32 (100%)	0 (0%)	0 (0%)	0 (0%)
	Surface texture (both groups)	32 (100%)	0 (0%)	0 (0%)	0 (0%)

**Table 7:** Deviation measurements.

Parameter		Neo spectra	Zenit	Test statistic	*p*-value	Rank-biserial correlation (95% CI)
Linear deviation (mm)	Mean ± SD	0.41 ± 0.15	0.60 ± 0.34	68.00	0.275	− 0.25 (− 0.61 to 0.18)
	Median (IQR)	0.43 (0.13)	0.47 (0.50)			
Volumetric deviation (mm^3^)	Mean ± SD	1.96 ± 1.83	1.38 ± 1.16	101.00	0.645	0.11 (− 0.32 to 0.50)
	Median (IQR)	1.42 (2.84)	0.95 (1.57)			

## Discussion

### Material chemistry

Dental professionals commonly use tooth-colored restorative materials to meet patients’ aesthetic demands. Since the advent of nanotechnology in dentistry, nanocomposites incorporating fillers ranging in size from 0.01 to 0.04 mm have been developed^18^. Nanoceramic composites, which are derived from ceramics through an organic transformation process, have recently been introduced. These materials feature a resin matrix embedded with nanofillers made of methacrylate-modified silicon dioxide, which is later replaced by a matrix containing highly dispersed methacrylate-modified polysiloxane nanoparticles. These nanoparticles, with a chemical composition similar to glass or ceramics, possess an inorganic structure akin to technical ceramics and offer exceptional biocompatibility^19^.

This randomized clinical study evaluates the performance of two composite materials (Zenit and Neo Spectra) using modified USPHS criteria, originally developed by Gunnar Ryge and later adapted by the California Dental Association to assess restoration quality based on acceptability rather than degrees of success. Selecting appropriate intervention and comparator materials is critical when designing clinical trials and comparative studies in healthcare research. The selection process involved thorough evaluation to ensure the materials’ clinical effectiveness and reliability in restoring Class I cavities in posterior teeth.

Neo Spectra utilizes a urethane dimethacrylate (UDMA)-based, Bis-GMA-free resin matrix, which significantly influences its mechanical properties, particularly wear resistance^20^. The characteristics of this matrix contribute to the composite’s durability under occlusal forces^21^. UDMA exhibits a lower intrinsic viscosity compared to Bis-GMA, facilitating a higher degree of polymerization^12^. This results in a more cross-linked polymer network, enhancing mechanical stability and wear resistance. Studies have shown that composites with UDMA-based matrices achieve a higher degree of conversion, leading to improved mechanical properties^22^.

The flexible nature of UDMA’s molecular structure allows for better absorption and distribution of occlusal stresses, reducing the likelihood of micro-crack formation and propagation^22^. This flexibility contributes to increased resistance to wear and fatigue. Research indicates that UDMA-based composites exhibit favorable flexural strength and fracture toughness, which are indicative of their ability to withstand functional loads^23^.

NeoSpectra™ ST incorporates organically modified ceramic nanoparticles and nanofillers alongside traditional glass fillers to enhance natural esthetics and reduce monomer release^23^. The Zenit nanoceramic composite (PRESIDENT DENTAL GmbH, Munich, Germany) features a matrix typically based on Bis-GMA (Bisphenol A-Glycidyl Methacrylate), a traditional monomer widely used in dental composites^24^. The characteristics of Bis-GMA directly influence mechanical behavior, including wear resistance^18^. Its high viscosity and rigidity contribute to strength but also result in a stiff polymer network with low flexibility. This rigidity can lead to the formation of brittle structures, making the composite more susceptible to microcracking under functional loads. Reduced energy absorption in such networks may also contribute to increased surface wear under occlusal forces^19^.

The current study demonstrates that the chemical composition of the resin matrix plays a crucial role in determining the clinical performance of dental composites. NeoSpectra™ ST, with its UDMA-based resin matrix, offers superior mechanical stability and wear resistance compared to traditional Bis-GMA-based matrices, such as that of Zenit. The lower viscosity of UDMA facilitates a higher degree of polymerization, contributing to a more cross-linked polymer network, which, in turn, enhances the material’s durability under occlusal forces.

### Wear patterns and measurement

Class I cavities were selected for this study to assess the wear behavior of resin composite materials. This choice was based on their consistent cavity morphology and direct exposure to occlusal forces, providing a reliable and reproducible framework for evaluating material performance under functional load. The straightforward nature of Class I cavities minimizes external variables, facilitating precise measurement of both surface and vertical wear. This focus enables an in-depth analysis of material-specific factors—such as resin matrix composition and filler morphology and their influence on wear resistance under clinically relevant conditions^25^.

In contrast, Class II cavities though also posterior restorations introduce additional clinical variables that can complicate the standardization of wear assessments^26^. These factors include variations in proximal contact, which can affect occlusal force distribution, as well as differences in margin placement, adjacent structures, and matrix systems^27^. Such complexities may hinder the consistency of wear evaluations and may not accurately reflect the intrinsic occlusal wear resistance of the material.

The increasing accessibility of intraoral 3D scanning in general dental clinics provides significant advantages for both patients and practitioners by enhancing efficiency, improving convenience, and enabling seamless data storage and sharing. This technology allows for precise wear assessment by superimposing sequential scans of teeth or jaws to quantify changes over time^25^. By employing a quantitative approach, it facilitates the measurement of tooth material loss in terms of both height and volume. However, accurate quantitative wear analysis requires advanced technological capabilities to ensure precision and reliability^28^.

Quantitative wear measurement in this clinical study was assessed using a novel iterative approach. Measurements were performed on the entire occlusal surface of each molar^5^. Superimposition of 3D datasets was conducted using the iterative best-fit alignment method, a technique widely employed in dental research for evaluating morphological changes over time. This method utilizes the Iterative Closest Point (ICP) algorithm to align sequential 3D scans by minimizing the distance between corresponding points on the surfaces, thereby achieving optimal alignment. The iterative best-fit approach provides a more automated and objective alignment process^29^. However, it is important to note that in regions exhibiting significant morphological changes, the algorithm may underestimate the extent of those changes due to its global minimization strategy. Despite this limitation, the iterative best-fit method remains a valuable tool in clinical studies, offering high-resolution, quantitative assessments of dental wear and supporting the evaluation of restorative materials’ performance over time^30^.

### Clinical performance

The modified USPHS criteria assessed eight parameters: anatomic contour, marginal adaptation, surface texture, gross fracture, marginal discoloration, color match, secondary caries, and postoperative sensitivity. According to the results of the present study, no statistically significant differences were observed between the two groups after 48 months, except for marginal discoloration. While some variations in clinical performance were noted at 48 months compared to baseline, these differences were not statistically significant (*p* = 0.112), including the amount of wear measured using intraoral scanning and Geomagic Control X 2022 3D superimposition software.

Therefore, the null hypothesis was accepted. Both restorative materials demonstrated acceptable performance, with no failures recorded during the study period. In terms of anatomic form, all restorations in both groups consistently received an alpha score at all evaluation intervals. The quantity of filler material directly influences surface hardness, which, in turn, affects wear resistance and the ability to maintain anatomical form^1^. The observed results could be attributed to the filler size and content (by weight/volume) of the tested materials. Since both composites analyzed in this study have comparable filler content and glass filler size, they likely exhibit similar hardness and surface characteristics^3^.

Regarding marginal adaptation, the findings revealed no statistically significant differences between the two groups throughout the observation period^31^. Regarding the marginal discoloration criterion, most restorations received Alpha scores, while Bravo scores were recorded only at the 48-month examination—four in the Zenit composite group and two in the Neo Spectra group. All cases of discoloration were confined to the enamel margin and were considered clinically acceptable. Marginal discoloration may indicate degradation of the interface between the restorative material and tooth structure, potentially leading to marginal leakage. This discoloration may be caused by food, smoking, or other external factors. A significant contributing factor is believed to be polymerization shrinkage, particularly in occlusal cavities with a high C-factor, which represents the ratio of bonded to unbonded surfaces^26^.

Regarding surface texture, the absence of a statistically significant difference between the two materials across different follow-up periods may be attributed to their filler composition. Finer particle sizes, combined with a higher filler volume-to-weight ratio, enhance resistance to external factors and minimize surface roughness. Additionally, spherical fillers exhibit lower roughness after polishing and chemical degradation compared to irregularly shaped fillers^32,33^.

Concerning gross fracture, the results indicated that all cases in both groups received Alpha scores throughout the follow-up periods. This may be attributed to the incorporation of fillers into the polymer matrix, which enhances fracture toughness, elastic modulus, and tensile strength^3^.

Concerning color matche, no statistically significant difference was observed between the two groups across the follow-up periods. This may be attributed to the staining behavior of the resin matrix in composite resins, as the lower water sorption and solubility of urethane dimethacrylate (UDMA) result in reduced discoloration compared to Bis-GMA or TEGDMA^27^.

Regarding secondary caries, the absence of detected cases across all tested restorative systems throughout the follow-up periods may be attributed to polymerization shrinkage and polymerization stress at the material/tooth interface^28^. In addition to the regular participation of most patients in a professional prophylaxis program once or twice a year, which likely contributed to a reduced risk of caries development. Sites where the explorer catches or encounters resistance upon removal—along with the presence of softness, opacity, etching, or white patches—are recognized as indicators of demineralization or structural compromise, facilitating the identification of secondary caries. Furthermore, bitewing radiographs (Kodak, Rochester, NY, USA) were obtained at each follow-up visit. However, a 48-month assessment may be insufficient for detecting secondary caries, as it typically manifests after four to six years of intraoral aging, as evidenced by previous long-term studies^26,27^.

Regarding postoperative sensitivity, the results showed that all cases in both groups received an Alpha score. Sensitivity was assessed for each restoration by sliding a probe over the restored tooth surface and applying a stream of compressed air for three seconds from a distance of two to three centimeters^26^.

Regarding wear, Zenit samples exhibited greater linear deviation than Neo Spectra, while Neo Spectra demonstrated higher volumetric deviation. However, neither difference was statistically significant. This may be attributed to the distinction between the two measurement types: linear deviation quantifies point-to-point depth changes, whereas volumetric deviation assesses overall material loss or gain in three-dimensional space. Opposing cusps that are flat or rounded tend to produce greater volumetric deviation, while pointed cusps generally result in greater linear deviation.

### Resin matrix and filler effects on wear

The non-significant difference between the two restorative materials may be attributed to their resin matrix composition, which significantly influences the wear resistance of resin composites. NeoSpectra™ ST incorporates a urethane dimethacrylate (UDMA)-based, Bis-GMA-free matrix, which facilitates a higher degree of polymerization and enhanced stress distribution^21^Zenit employs a traditional Bis-GMA-based matrix characterized by greater rigidity and lower flexibility^24^. Despite these differences, both materials exhibited comparable wear resistance, which may be attributed to their closely matched filler content NeoSpectra™ ST with 78–80% by weight (57% by volume) and Zenit with 83% by weight (70% by volume). Increased filler volume-to-weight ratios are associated with improved wear resistance and reduced surface roughness under occlusal loading.

In addition, particle size distribution plays a critical role in wear behavior^3,33^. Zenit contains finer fillers (0.7 μm) compared to NeoSpectra™ ST (5 μm), which contributes to a denser filler network, minimizing interstitial spaces and improving the structural integrity of the composite^22^. This dense filler architecture reduces both the depth and volume of wear over time. Furthermore, both materials maintained similar surface textures and anatomical form throughout the 48-month observation period, suggesting that filler hardness and volume—key factors in long-term wear resistance—were adequately optimized in both systems^1,3^, thereby explaining the absence of statistically significant differences in their wear performance.

### Clinical implications

These findings have important clinical consequences. Although both restorative materials performed well during the evaluation period, Neo Spectra may be a superior choice in clinical practice, especially in cases where long-term marginal esthetics are critical, because it displayed more consistent performance in terms of marginal discoloration. Furthermore, Neo Spectra demonstrated better handling characteristics during clinical application, which may support its selection. Zenit remains a clinically acceptable alternative, with comparable wear resistance, and may still be considered based on specific case requirements or clinician preference.

### Limitations of the study

Wear can be quantified using depth, area, and volume^5^. To assess the level of wear in different situations, most research investigations have utilized three-dimensional datasets of study models^15^. However, alterations in the dimensions of materials can potentially impact precision, particularly when dealing with measurements at the micrometer scale^34^. The acquired three-dimensional datasets were subsequently analyzed using various software programs.

To compare 3D datasets accurately, it is crucial to apply an alignment technique that results in a rigid transformation, optimally adjusting the two models^35^. The choice of alignment method has a substantial impact on the measurement outcome. Unlike reference-based alignment, best-fit alignment minimizes the mesh distance error between datasets but may underestimate the magnitude of change in the area of interest^30^. Nonetheless, the oral cavity lacks a stable reference structure, making best-fit alignment a practical alternative in clinical studies.

One limitation of this study is that wear data were given as absolute values instead of being normalized to the original restoration volume or standardized according to molar type. Even though all the restorations possessed similar Class I cavity configurations and were analyzed with a uniform digital protocol, anatomical differences between permanent molars could introduce subtle differences in occlusal load and surface area.

Despite the advantages of using an intraoral scanner (IOS) for non-invasive, high-resolution wear assessment, this study acknowledges several limitations associated with the digital workflow. First, the virtual models generated by IOS are susceptible to minor inaccuracies due to inherent digitization artifacts, which can affect the precision of volumetric and linear wear measurements,4 Additionally, the accuracy of 3D superimposition is highly dependent on software parameters, such as alignment algorithms and the selection of reference areas. Variability in these parameters may lead to inconsistent results, particularly in regions lacking stable anatomical landmarks. To minimize such variability, all scans in this study were performed under standardized environmental conditions, using the same operator and equipment, and in strict accordance with the manufacturer’s protocol.

Another critical factor influencing IOS accuracy is the presence of intraoral moisture, particularly saliva. Salivary contamination can interfere with image acquisition and compromise scan quality^7^. In the current study, strict moisture control was implemented using lip retractors, absorbent pads, and high-volume suction during all scanning procedures. However, the potential influence of residual moisture cannot be entirely excluded.

Best-fit superimposition has inherent limitations even though it offers a very accurate way to evaluate surface wear. The possible underestimation of wear in morphologically altered or mobile areas (particularly cusp tips and occlusal contact points) is a major worry. If nearby anatomical landmarks have changed or deformed over time, localized wear may be averaged out or distorted because best-fit algorithms align datasets by minimizing overall surface deviations. This restriction may marginally impair the accuracy of volumetric wear detection in regions with high occlusal stress and is especially pertinent to clinical investigations involving natural functional loading.

Wear measurements may be impacted by minute differences in scanning technique or choices made during data alignment and processing, even though the scans were carried out by a single calibrated examiner adhering to established protocols. To reduce this bias, future research could benefit from evaluating inter-operator reliability and including multiple operators.

Furthermore, while IOS offers considerable advantages for chairside applications and longitudinal monitoring, its precision remains inferior to that of laboratory-grade optical scanning systems. These limitations must be considered when interpreting the results and may partially explain subtle discrepancies or variances in quantitative wear measurements.

The 48-month follow-up may not be enough to identify the emergence of secondary caries or late-stage restoration failures, even though it offers useful mid-term data on the clinical performance and wear of nanoceramic composites. To properly evaluate the long-term durability and clinical success of these materials, more extensive longitudinal studies are necessary because these results frequently appear over longer time periods.

## Conclusions

Within the limitations of this study, the following conclusions can be drawn:

1. Both NeoSpectra™ ST (UDMA-based) and Zenit (Bis-GMA-based) composites demonstrated clinically acceptable performance over a 48-month period in Class I posterior restorations.
2. Some marginal discoloration was observed with both materials.
3. The differing matrix-to-filler ratios of the two nanoceramic resin composites may have contributed to compensating for volumetric wear.
4. The wear patterns differed between the materials: linear deviation was greater in Zenit, while volumetric deviation was greater in NeoSpectra™ ST.
5. Quantitative wear measurement using intraoral scans offers an accurate and reliable method independent of operator experience. The integration of IOS technology with 3D surface-based superimposition techniques demonstrates high efficacy in the early detection and monitoring of tooth or restorative material wear.

## Recommendations

1. Clinical studies with longer follow-up periods should be conducted to verify the current findings and evaluate clinical performance over an extended duration of service.
2. It is recommended to perform clinical trials to assess the effectiveness of Zenit nanoceramic resin composite compared to other restorative materials in compound and complex cavities.
3. The precision of intraoral scanners should be sufficient to detect and monitor tooth wear, as studies suggest that comparing individual teeth or smaller regions—rather than full arches—enhances the accuracy of quantitative wear measurements.
4. The new Medit intraoral scanner, equipped with Medit Link software and its Deviation Display feature, has the potential to accurately capture three-dimensional images and assess quantitative tooth wear.
5. When interpreting data on tooth material loss, it is important to consider the measurement error and inherent uncertainty associated with this method.

### Clinical significance

1. The clinical performance and occlusal wear behavior of Zenit Nano-Ceramic Resin Composite (Bis-GMA-based) and Neo Spectra™ ST Nano-Ceramic Resin Composite (Bis-GMA-free) were similar, and both were considered clinically acceptable.
2. Clinicians can anticipate longer-lasting restorations with less wear and fewer failures when selecting materials with optimal resin chemistry and filler integration.
3. The application of intraoral scanning (IOS) for non-invasive wear assessment provides an advanced method for the systematic monitoring of restorative materials. When coupled with 3D superimposition technology, this approach enables dental professionals to precisely evaluate wear patterns and structural alterations in restorations over time. Such high-accuracy monitoring facilitates optimized treatment planning and enhances predictive outcomes, ultimately improving patient care and long-term restoration performance.

## Data Availability

The data that support the findings of this study are available from the corresponding author, on reasonable request.

## Abbreviations

Modified USPHCs: Modified United State Public Health Criteria
P: Probability of chance
RBCs: Resin-based composites
HEMA: 2-Hydroxyethyl methacrylate
IOS: Intra-oral scanner
PENTA: Di pentaerythritol pent acrylate phosphate
10-MDP: 10-Methacryloyloxydecyl dihydrogen phosphate
Bis-GMA: Bisphenol-A-diglycidyl methacrylate
TEGDMA: Triethyleneglycol dimethacrylate
UDMA: Urethane dimethacrylate
1PENTA: Di pentaerythritol pent acrylate phosphate
